# Determinants of Malaria Prevention and Treatment Seeking Behaviours of Pregnant Undergraduates Resident in University Hostels, South-East Nigeria

**DOI:** 10.1155/2017/3653874

**Published:** 2017-12-03

**Authors:** Anthonia Ukamaka Chinweuba, Noreen Ebelechukwu Agbapuonwu, JaneLovena Enuma Onyiapat, Chidimma Egbichi Israel, Clementine Ifeyinwa Ilo, Joyce Chinenye Arinze

**Affiliations:** ^1^Department of Nursing Sciences, University of Nigeria, Nsukka, Enugu Campus, Enugu, Nigeria; ^2^Department of Nursing Science, Nnamdi Azikiwe University, Nnewi Campus, Nnewi, Nigeria

## Abstract

This cross-sectional descriptive survey investigated determinants of malaria prevention and treatment seeking behaviours of pregnant undergraduates resident in university hostels, South-East Nigeria. Purposive sampling was used to enrol 121 accessible and consenting undergraduates with self-revealed and noticeable pregnancy residing in twenty-three female hostels of four university campuses in Enugu State, Nigeria. Structured interview guide developed based on reviewed literature and WHO-recommended malaria prevention and treatment measures was used to collect students' self-report data on malaria preventive health behaviours, sick role behaviours, and clinic use using mixed methods. The WHO-recommended malaria prevention measures were sparingly used. Some believed that pregnancy does not play any role in a woman's reaction to malaria infection. Only 41 (50.6%) visited a hospital for screening and treatment. Thirty-four (28.1%) used antimalaria medicine bought from chemist shop or over-the-counter medicines, while 33 (27.3%) used untreated net. The students were more likely to complete their antimalaria medicine when they were sick with malaria infection than for prevention (*p* = 0.0186). Knowledge, academic schedule, cultural influence on perception and decision-making, and accessibility of health facility were key determinants of the women's preventive and treatment seeking behaviours. Health education on malaria prevention and dangers of drug abuse should form part of orientation lectures for all freshmen. University health centres should be upgraded to provide basic antenatal care services.

## 1. Introduction

Malaria in pregnancy remains a public health challenge due to its related prenatal mortality. Its impact is notably high in malaria endemic areas like Nigeria. “About 50% of Nigerian population have at least one episode of malaria each year with nearly 110 million clinical cases and an estimated 300,000 deaths per year” [1, 2]. “Women and children are the vulnerable groups” [3, 4]. Schantz-Dunn and Nour [5] observed that “women were three times more at risk of suffering from severe effects of malaria in the pregnant than in the non-pregnant state and that women who had malaria infection during pregnancy have higher risks for complications of pregnancy including neonatal and maternal deaths.”

Available studies already indicate an “increased prevalence of malaria infection among Nigerian university students residing in school hostels” [6, 7]. “These school hostels were ill-equipped and their environmental conditions poor” [8–11]; “and, the school healthcare facilities are characterised by inadequate manpower, out-of-stock syndrome and poor quality healthcare” [12, 13]. Some of these students are pregnant women who are even more vulnerable to the adverse effect of malaria infection and need to take extra precaution in preventing and treating the infection. This study investigated the determinants of malaria prevention and treatment seeking behaviours of pregnant undergraduates resident in school hostels in Nigeria. The purpose of the study, therefore, was to assess the factors related to the pregnant undergraduates' preventive health behaviour, sick role behaviour, and their clinic use in prevention and treatment of malaria. Specifically, the study determined whether the women were aware of their risk for increased complications of malaria in their pregnancy state, actions they took to prevent malaria attack during pregnancy, what they did to treat malaria when they felt they had it, under what conditions they sought medical care for malaria treatment, where they accessed care for prevention and treatment of malaria and why, and how compliant they were in use of prescribed antimalaria medicines.

Malaria is known to be preventable and curable. However, “malaria infections during pregnancy are mostly asymptomatic [14].” Pregnant students in school hostels are expected to take appropriate actions to prevent the disease. Prevention targets the vector (mosquito), the reservoir (human host), and the causative agent (the* Plasmodium*). WHO [15, 16] recommends “a number of malaria prevention strategies including: use of personal protective measures such as long-lasting insecticidal-treated bed nets (LLINs), covering exposed skin to prevent mosquito bites, screening of houses; environmental modification which involves creating drains to prevent swamps, covering any stagnant water, clearing vegetation from water bodies, effective rubbish disposal, spraying insecticides on breeding habitats, growing mosquito repellant plants like Neem tree; vector management using insecticides such as in-door residual spraying (IRS) and use of mosquito repellants; and, intermittent preventive treatment in pregnancy (IPTp) with sulfadoxine-pyrimethamine (SP) (particularly for pregnant women in malaria-endemic areas).”

Several studies had been done on prevention and treatment of malaria in pregnancy in the Nigerian society. Onyeneho et al. [17] studied perception and attitudes of pregnant women in Enugu State, Nigeria, towards prevention of malaria infection. Result showed that some of the women were ignorant of what to do to prevent malaria infection. Anumudu et al. [18] assessed the treatment seeking behaviour of 307 young students of University of Ibadan, Nigeria. They observed that ITN and IRS were sparingly used as malaria prophylactics; almost half of the respondents were self-medicated while more than half did nothing. Okwa and Ibidapo's [19] study on the malaria situation, perception of cause, and treatment in a Nigerian University yielded similar result. Pregnant students living in school hostel have received few research attention, hence this study.

## 2. Methods

The design was cross-sectional descriptive survey. The target population was all pregnant undergraduates residing in twenty-three (23) female hostels of four university campuses in Enugu State, Nigeria (designated A–D for confidentiality). However, as the exact population could not be determined (infinite population), incidental/purposive sampling was used to enrol all undergraduates with self-revealed and noticeable pregnancy residing in the university hostel into the study. Based on this, 51 pregnant undergraduates were recruited from 10 hostels in “A” university, 27 from 6 hostels of “B” university, 18 from 2 hostels of “C” university, and 32 from 5 hostels of “D” university. One hundred and twenty-eight (128) accessible and consenting pregnant undergraduates, thus, were recruited for the study. Seventeen of these hostels are within the school premises while six are located at trek distances to, but owned by, the school.

### 2.1. Instrument

The instrument used for data collection was 10-item structured interview guide developed by the researchers based on reviewed literature and WHO-recommended malaria prevention and treatment measures profile. Four items were on the students' personal profiles of age, gravidity, year of study, and gestational age. Three open-ended items collected qualitative data on whether they were aware of their risk for increased complications of malaria in their pregnancy state; reasons for measures they adopted to prevent and treat malaria; and condition(s) under which they sought medical care for malaria treatment.

Three close-ended items were on what they do to prevent and treat malaria infection, whether they had malaria in present pregnancy, and their use of prescribed medication for malaria prevention and treatment. Malaria preventive measures include actions employed to avoid mosquito bites and malaria attack such as use of ITN, mosquito repellents or electric mosquito swatter, IRS, and IPT, while malaria treatment measures include measures taken during an attack of malaria to contain the disease, for example, seeking treatment in a health facility or first line treatments.

The instrument was pilot-tested by administering it to twelve undergraduates resident in two female hostels of a university in Anambra State, Nigeria (about 10% of the sample), which is in the same political zone and similar in characteristics to universities of the study population. Data collected were subjected to Cronbach alpha reliability test. A coefficient alpha of 0.81 and a standardized item (inter item) coefficient of 0.87 were obtained.

### 2.2. Ethics Approval

Ethical clearance for the study was obtained from the Research Ethical Committee of University of Nigeria Teaching Hospital, Ituku-Ozalla, Enugu, Nigeria (Ref. number UNTH/CSA/329/VOL.5). Administrative permit was also obtained from the appropriate university authorities, Student Affairs Unit heads, and hostel supervisors. Prior to data collection, prospective respondents were approached and purpose of the study carefully explained. Only resident students who gave their consent after due explanation were used.

### 2.3. Instrument Administration

Four (4) research assistants comprising one hostel student-leader (hostel governor) in each of the four universities were recruited and used for data collection after 30–60-minute discussion of objectives of the study, contents of the instrument, selection of subjects, how to administer the interview guide for data collection, and interpretation of the questions in the instrument (where needed). Objectivity and confidentiality on information gathered were emphasized.

One researcher visited the hostels of one university in the company of research assistant from that university. Using the snowball method, the researcher or assistant identified one pregnant student residing in university hostel in each of the four universities with the help of the hostel warden after administrative permit had been obtained. Researcher or assistant introduced self and purpose of the study. After obtaining oral consent, the respondent was engaged in face-to-face interview with the researcher or assistant on her malaria prevention and treatment behaviours. The interview process was guided strictly by contents of the interview guide. After the interview, the respondent was requested to identify another pregnant student in the same hostel using room number for easy access. Data collection was between 6.00 p.m. and 8.00 p.m. from Mondays to Thursdays when many of the students were likely to be in their rooms. Repeat visit was made where necessary for administration of the interview. The data collection lasted for six weeks and three days.

### 2.4. Method of Data Analysis

Quantitative data collected on demographic characteristics reported malaria infection and measures used for malaria prevention and treatment were analysed descriptively using frequencies, percentages, mean, and standard deviation. Contingency table on association between student's health behaviour on use of prescribed medication and purpose of the medication intake was subjected to Fisher's exact test. Qualitative data on respondents' awareness of their risk for increased complications of malaria in their pregnancy state and reasons for their specific actions towards malaria prevention and treatment were transcribed to form concepts. The emerging concepts were categorized based on the study objectives, coded, and subjected to conventional content analysis using template approach. Seven themes emerged. The statistical analyses were performed using a biostatistical software, the statistical package for social sciences (SPSS) version 20.0 computer software programme (SPSS inc., Chicago, IL, USA) at 95% confidence interval. Data collected on reasons for “actions taken” and “no special action” were qualitatively subjected to content analysis to form themes.

## 3. Results

Out of the 128 copies of the instrument administered, 121 were retrieved giving a return rate of 94.5%. Results are shown in Table 1.

Sixty-seven (55.4%) of the respondents were in the last two months of their pregnancy, while 12 (10.0%) had pregnancy below 6 months. There was no definite pattern in age distribution of the women in relation with their gestational age. However, women aged 21–25 were the highest (51), 29 (24.0%) of whom were more than 8 months pregnant. The least was one (0.7%) student aged 16–20 with pregnancy under 6 months. Majority of the women, 89 (*73.6%),* were primigravida, 53* (43.8%)* of whom were more than 8 months pregnant. Forty-nine (*40.5%)* were in their fourth year of study; first year students were the least 4 (*3.3%)*. There was no definite pattern in the distribution of the students in terms of their age of pregnancy (Table 1).

Majority, 81 (66.9%), of the resident students that entered the study reported that they have had malaria in pregnancy. Sixty-six (74.2% of the 89 primigravidae) and 13 (50.0%) of the 26 secundigravidae had malaria but only 2 (33.3%) of the 6 women pregnant for the third time or more had malaria. In all, the primigravidae reported malaria infection more than the rest of the women (Table 2).

### 3.1. Preventive Health Behaviours

*Whether the Women Were Aware of Their Risk for Increased Complications of Malaria in Their Pregnancy State*. Respondents were asked to give their opinion on the statement that “malaria can cause more health problems to the pregnant woman and her unborn baby than the non-pregnant woman.” Result showed some attribute malaria to other factors which they found in both pregnant and nonpregnant women. The women believed that pregnancy does not play a role in a woman's reaction to malaria attack. Such women stated that* “*it's only those who do not eat very well that can be seriously affected by malaria, pregnancy neither increases the risk of malaria nor does malaria increase the risk of other problems in pregnancy”; “it depends on the person's genotype, not pregnancy”; or that “women who take too much oil or fatty foods suffer a lot from malaria, whether pregnant or not. It can also make the baby to be too big.”

One said “well, pregnant women should avoid working for a long time under the sun because this is one major cause of malaria.” Some pointed out that factors other than malaria were actually responsible for complications of pregnancy the woman may experience. Some of their comments in response to researchers' statement include: “It is true that malaria is more common in women but pregnancy does not increase the chances of women having more problems from malaria”; “Mosquito bite can cause malaria but cannot kill the baby in the woman's womb”; “It is true that malaria is dangerous for pregnant women but as Africans, it is rarely as deadly as books will present it to be”; and “Malaria does not affect an unborn child because mosquito cannot bite him in the mother's womb, therefore, both pregnant and non-pregnant women suffer the same.”

Some, though few, however agreed that malaria is capable of causing poor pregnancy outcome. Some of their statements were, “Yes, it is possible because malaria makes one to lose appetite but a pregnant woman is supposed to eat very well to be strong and the baby to grow very well”; “A woman that is not pregnant can take any medicine to treat her malaria but pregnant women are not allowed to take medicines anyhow. Delay in taking treatment can, therefore, cause death of the baby”; and “Fever the pregnant woman has during malaria attack can pass to the baby and kill him.”

The result in Table 3 showed that, for vector control and prevention, 40 (33.1%) of the students would always avoid stagnant water in open containers in their rooms. For prevention of reservoirs, 33 (27.3%) slept under untreated nets. However, only 16 (13.2%) had their nets treated with insecticide always. Twenty-eight (23.1%) said they protect themselves from mosquito bites by always wearing protective clothing. To prevent the effect of plasmodium, only 25 (20.7%) would always use medicines they collected from hospital/clinic while 34 (28.1%) always used antimalaria medicine they bought from the chemist shop without prescription or over-the-counter medicines (OTC).

Overall, indoor residual sprays appeared to be the most frequently used preventive measure, used always by 32 (26.5%) and sometimes by 63 (52.1%) (mean = 2.1; SD = 0.69), while the least was use of mosquito coils to ward off mosquitoes (mean = 1.2; SD = 0.48). Other (volunteered) preventive actions were use of mosquito squatter to catch and kill mosquito and prevention of infection using nutraceuticals by 50 and 4 students, respectively.

### 3.2. Sick Role Behaviours

Respondents were asked to indicate, from the listed options, the action(s) they took to prevent and/or treat malaria infection. Provision was also made for additional information not contained in the list.  Table 4 presents the responses obtained. Items in italics were additional data as provided by the respondents. Result showed that only 39 (32.2%) of the pregnant undergraduates take actions to prevent malaria, while 81 (66.9%) took varied actions to treat the disease. The rest would not take any action. Many would take some medications to prevent malaria or when they feel they have the infection, although the source and type varied. Only 41 (50.6%) would visit the hospital for screening and treatment, while 16 (41.0%) do so to prevent malaria. Respondents resorted to self-medication more frequently than they would visit hospital; hence, as many as 51 (63.0%) bought any antimalaria medicine available at a chemist shop for treatment. Some resorted to local herbs in common use at their home for treating malaria; even in pregnancy, for instance, 2 (5.1%) took the fresh neem leaves juice* (Azadirachta indica) *for malaria prevention and 9 (11.1%) for treatment. Five (12.8%) undergo the traditional steam bath ritual, the “okpukpu aju,” to treat malaria. Seven (8.6%) took analgesics to treat malaria.

The women were asked to indicate whether they took complete dose of their prescribed antimalaria medicines for purpose of prevention and/or treatment of malaria. Result showed that as many as 46 (85.2%) of the women would complete their prescribed medication when sick with malaria infection while 10 (55.6%) would do so to prevent the infection. Fisher's exact test result showed significant difference in the respondents' health behaviour on use of prescribed medication and purpose of the medication intake (*p* = 0.0186) (Table 5). 

*Reasons for Specific Actions towards Malaria Prevention and Treatment*. Thematic presentation of summary of reasons given by the respondents for their specific actions towards malaria prevention and treatment include the following.

*Seek Healthcare in the Hospital*. Some respondents, particularly those in health-related field of study, took actions because they understood how dangerous malaria is and as one added, “did not want to have a bit of it.” They also wanted measures that would not cause other problems/diseases to them. A respondent said, “Malaria makes me seriously sick so I will do everything possible to avoid it.”

*Self-Medicate*. Ignorance seems to play a major role here. The general impression of the respondents is summarised by a response, thus: “there are many anti-malaria medicines available in the chemist shop and I know that anti-malaria medicines are important in pregnancy so I do not need the hospital to know what to take for malaria treatment.” Others said:,*“*Yes, I know that malaria is dangerous in pregnancy but I have no time to go for antenatal clinic so I buy any available anti-malaria medicines.” Several numbers of respondents said they resort to self-medication because there was no hospital or clinic providing antenatal services for pregnant women near their hostel. Some expressed their willingness to visit a hospital outside the school but were discouraged by thought of the long protocols and waiting time in hospitals/clinics, “so I go to where it will be fast for me; I need time for my lectures, you know!” one concluded. These findings showed that some merely choose a measure because it is convenient to them while others apply measures based on their perceived susceptibility and severity of the illness. 

*Use Local Herbs*. Some still hold tight to their traditional way of treating illness even in pregnancy claiming that there are some safe, cheap, and effective herbs commonly used to treat malaria in their community. They, therefore, prefer to go for these local herbs. One eighteen-year-old primigravida in her “eighth month of pregnancy” declared that she would break her education at her ninth month. She said, “I will go home to my mother to get some local herbs to cleanse my baby,” “I treat any disease like malaria with herbs my mother will prepare for me”, and “I will continue with the medicine until I deliver my baby at home; no need for hospital.” 

*Prayer*. Two respondents clearly stated that they do not need medicine to “*receive*” healing; they only pray because “only God heals.” One said she usually prays but when the problem gets worse, she can then go for treatment as last option.

*No Special Action*. Reasons given for not taking any action to prevent and treat malaria include perceiving malaria as a minor illness, that is, a non-life-threatening illness. A respondent vividly said, “In Africa, malaria is a common problem and usually goes without treatment, so I do not need to give it serious attention.” Some of the reasons for no action against malaria were related to lack of time to deal with it, like those who would love to go to hospital but for the protocols; hence one said, “I think less of how to prevent malaria because I have a lot of academic tasks.” There is also a cost implication; that is, availability of fund determines the choice of malaria prevention and treatment measures, as one said, “I use methods (measures) I can afford.” Some of the women may not be able to procure the ITN due to financial constraints.

### 3.3. Clinic Use

For conditions under which respondents sought medical care for malaria treatment, the forty-one (41) respondents who visited a health facility for treatment were further requested, using an open-ended question, to specify the condition(s) under which they took decision to seek medical aid in hospital. Their responses were coded, categorized, and subjected to conventional content analysis using template approach. Five themes emerged from the analyses. Result showed that 22 sought medical aid only when they were very ill. Nine sought medical care with the slightest feeling of ill-health. Four went to the health facility when it was time for their antenatal clinic, while the rest visited the hospital when their condition got worse.

## 4. Discussion

### 4.1. Preventive Health Behaviour

Result showed that although malaria is endemic in Nigeria; the respondents had varied perceptions of the severity of malaria in pregnancy and their susceptibility. This is a significant determinant of actions they took against the disease. The perception of “no difference in the effect of malaria in the pregnant and non-pregnant state” and the assumption that “pregnancy does not play a role in a woman's reaction to malaria attack” by some of the students means that they may not take any special precaution during their pregnancy period to prevent malaria and its possible complications. In fact, this was obvious when as many as 82 (67.8%) said they took no special action to prevent malaria in their pregnancy. Similarly, the erroneous impressions that malaria is not possible cause of complicated pregnancies, but other factors such as “hard labour under the sun for a long time” and “intake of too much fatty food” and that “there is no relationship between pregnancy and severity of malaria,” suggest that cultural beliefs still play palpable roles in preventive health behaviour and sick role behaviour of the population. This may be related to the findings of Anumudu et al. [18] which revealed that more than 50% of their respondents did nothing to prevent malaria attack. These findings, therefore, further strengthen the need for more robust enlightenment programmes on effect of malaria on pregnancy and malaria prevention and treatment especially in pregnancy in malaria endemic areas.

The WHO-recommended preventive measures were sparingly used. Although WHO [15, 16] advocate the use of ITN, very few seemed to find this relevant even in their pregnancy state as only 6.6% used ITN always; 27.3% used nets, but untreated, undermining the importance of the insecticide. The most frequently used malaria preventive measure among the respondents, though by few, was IRS, used always by 32 (26.5%). This is more evident in the finding that less than half of the subjects seemed to be aware of the effect of plasmodium on their pregnancy and few would use antimalaria medicines collected from hospital/clinic or chemist shop. This finding is thus in contrast to Okwa and Ibidapo [19]. In all, the students' preventive actions were suboptimal since the grand mean for each of the three malaria prevention strategies was <2. This implies that many of the students have poor knowledge of impact of malaria in pregnancy and measures for preventing it, thus supporting the findings of Onyeneho et al. [17]. Ordinarily, one would think that the students' exposure to teachings on environment-health related diseases like malaria through sources including classroom lectures, books, internet, and the media would improve their understanding of malaria and appropriate malaria preventive health behaviours. This assumption was, however, not reflected in the findings.

### 4.2. Sick Role Behaviour and Clinic Use

The students seem to be not proactive in handling malaria in pregnancy. Results of the study showed that the women were more likely to complete their antimalaria medicine when they were down with malaria attack than when the medicine was taken for preventive purposes. The result further suggests that the students had poor knowledge of risks associated with self-medication, particularly in their pregnancy state. They could, therefore, walk into a chemist shop and buy any antimalarial medicine to prevent (24.8%) or treat (42.2%) malaria. It was also noted that more of the subjects would self-medicate than go to hospital for treatment when they feel they have malaria except when they had serious symptoms of the illness. No wonder why they indulged in self-medication (30 (24.8%)) rather than using hospital services (16 (13.2%)) for prevention of malaria in pregnancy. This finding has implication for law on medicine sale, procurement, and use for treatment of diseases in Nigeria, which may be considered weak. Coincidentally, because malaria is common in Nigeria, there are many brands of easily accessible antimalaria medicines in the market. These women could freely walk into the medicine shop and buy any available brand of their choice to save time and cost since they self-medicate because “that was the best and fastest available option” for them. This practice exposes the fetus to drug-related complications. It also constitutes high risk to the populace because fake and adulterated drugs may be consumed resulting to health problems that may be fatal. There is also the possibility that lack of antenatal care services in the school health facility, as reported, is responsible for the poor clinic use.

Although majority took medicines when they had malaria, only about 33.9% visited the hospital for screening and treatment. It is however possible that those who sought care from hospital did so because of their “inexperience” and anxiety since the majority were young primigravidae. Also, they were more likely to complete their antimalaria medications when they were “really down with malaria attack” than for preventive purposes. The fact that only twenty-two used health facilities when they were down with malaria is worrisome and spells no good for efforts toward achievement of the third sustainable development goal (SDG_3_), good health and well-being. Surprisingly, as many as 33.1% admitted that they did nothing when they felt they had malaria, even in pregnancy, showing the enormity of their ignorance. This finding is, however, less than the 50% observed by Anumudu et al. [18] and Okwa and Ibidapo [19] in their previous studies, respectively.

The implication of these findings is that, even in the presence of available malaria prevention and treatment options as provided by the WHO, a good number of the students are still at risk of malaria-related complications of pregnancy and poor pregnancy outcome. The findings also revealed that majority of the students practice self-medication to treat malaria, which poses a challenge to effective malaria treatment. Thus, there is need for increased sensitization on the dangers of antimalaria self-medication.

## 5. Conclusion

Findings revealed four basic determinants of the pregnant students' health seeking behaviour: the women's knowledge of risks associated with malaria in pregnancy; cultural influence on perception and decision-making; availability of dedicated healthcare services in schools; and academic schedule. Ignorance, negative cultural beliefs, busy academic schedule in the absence of policy that protects the interest of pregnant students, and lack of access to quality health services are, therefore, implicated in the women's poor preventive and treatment health seeking behaviours. They are rarely proactive in preventing and treating malaria in pregnancy. They use the available school health services sparingly, even when they have symptoms of malaria. IRS and netting are frequently used while self-medication with antimalaria medicines were most common treatment measure.

## Figures and Tables

**Table 1:** Respondents' characteristics and whether they have had malaria in their pregnancy *n* = 121.

Demographic characteristics		Gestational age		
	*n* (%)	<6 months (12, 10.0%)	6–8 months (42, 26.5%)	>8 months (67, 55.4%)
Age *(years)*				
16–20	39* (23.2%)*	1 *(0.8%)*	14 *(11.6%)*	24 *(19.8%)*
21–25	51 (*42.2%)*	5 *(4.1%)*	17 *(14.1%)*	29 *(24.0%)*
26–30	19 (*15.7%)*	3 *(2.5%)*	7 *(5.8%)*	9 *(7.4%)*
≥31	12 (*10.0%)*	3 *(2.5%)*	4 *(3.3%)*	5 *(4.1%)*
Gravidity				
Primigravida	89 (*73.6%)*	2 *(1.7%)*	34 *(28.1%)*	53 *(43.8%)*
Secundigravida	26 (*21.5%)*	7 *(5.8%)*	7 *(5.8%)*	12 *(10.0%)*
Gavida ≥ 3	6 (*5.0%)*	3 *(2.5%)*	1 *(0.8%)*	2 *(1.7%)*
Year of study				
First year	4 (*3.3%)*	1 *(0.8%)*	3 *(2.5%)*	0 *(0%)*
Second year	14 (*11.6%)*	3 *(2.5%)*	4 *(3.3%)*	7 *(5.8%)*
Third year	31 (*25.6%)*	2 *(1.7%)*	15 *(12.4%)*	14 *(11.6%)*
Fourth year	49 (*40.5%)*	4 *(3.3%)*	13 *(10.7%)*	32 *(26.5%)*
Fifth year	23 (*19.0%)*	2 *(1.7%)*	7 *(5.8%)*	14 *(11.6%)*

**Table 2:** Cross-tabulation of gravidity and gestational age with incidence of malaria in present pregnancy.

Gravidity	Gestational age							
	<6 months		6–8 months		>8 months		Total	
	*n*	Had malaria	*n*	Had malaria	*n*	Had malaria	*n*	Had malaria
Primigravida (89)	2	2 *(100%)*	34	20 *(58.8%)*	53	44 *(83.0%)*	89	66 *(74.2%*
Secundigravida (26)	7	3 *(42.9%)*	7	7 *(100%)*	12	3 *(25.0%)*	26	13 *(50.0%)*
Gravida ≥ 3 (6)	3	1 *(33.3%)*	1	0 *(0.0%)*	2	1 *(50.0%)*	6	2 *(33.3%)*

*Total*	**12**	**6 *(5.0%)***	**42**	**28* (21.1%)***	**67**	**47 *(38.8%)***	**121**	**81 *(66.9%)***

**Table 3:** What respondents do to prevent malaria infection.

Malaria preventive measures	Always	Some times	Never	Mean
	3	2	1	(SD^*∗*^)
Vector *n* *(%)*				
Use indoor residual sprays	32 * (26.4)*	63 * (52.1)*	26 * (21.5)*	2.1 (0.69)
Use mosquito coils to ward off mosquitoes	4 * (3.3)*	12 * (9.9)*	105* (86.8)*	1.2 (0.48)
Avoid stagnant water in open containers in room	40 * (33.0)*	11 * (9.1)*	70 * (57.9)*	1.8 (0.91)
Reservoir *n (%)*				
Sleep under insecticide-treated net	8 * (6.6)*	10 * (8.3)*	103* (85.1)*	1.4 (0.70)
Sleep under net but not treated with insect repellants	33 * (27.3)*	25 * (20.7)*	63 * (52.0)*	1.8 (0.86)
Screen door and windows with net	23 * (19.0)*	3 * (2.5)*	95 * (78.5)*	1.4 (0.79)
Use mosquito repellent creams/oil	18 * (14.9)*	24 * (19.8)*	79 * (65.3)*	1.5 (0.74)
Wear protective clothing	28 * (23.1)*	17 * (14.1)*	76 * (62.8)*	1.6 (0.84)
Causative agent *n (%)*				
Use medicine collected from hospital/clinic	25 * (20.7)*	20 * (16.5)*	76 * (62.8)*	1.6 (0.81)
Buy antimalaria medicine from chemist shop (or OTC)	34 * (28.1)*	40 * (33.1)*	47 * (38.8)*	1.9 (0.82)
Take medicinal herbs from home	14 * (11.6)*	18 * (14.9)*	89 * (73.5)*	1.4 (0.69)
*Other actions*				
Use electric mosquito swatter	25 * (20.7)*	35 * (28.9)*		
Use nutraceuticals	3* (2.5)*	1* (0.8)*		

SD^*∗*^ = standard deviation.

**Table 4:** Actions the respondents take to prevent and treat malaria infection.

Action to prevent and/or treat malaria	Purpose of action	
	To prevent malaria (*n* = 39, 32.2%)	To treat malaria (*n* = 81, 66.9%)
Go to hospital	16 (41.0%)	41 (50.6%)
Buy any antimalarial medicine at chemist shop	30 (76.9%)	51 (63.0%)
Use local herbs/home remedies		
*Fresh neem leaves juice*	2 (5.1%)	9 (11.1%)
*Scent leaves*	3 (7.7%)	—
*Magic kola*	4 (10.3%)	2 (2.5%)
*Bitter kola*	1 (2.6%)	3 (3.7%)
*Bitter leaf juice*	—	—
*Undergo steam bath ritual* “(okpukpu aju)”	5 (12.8%)	—
*Take some analgesics to relief symptoms*	—	7 (8.6%)
Take nutraceuticals	4 (10.3%)	—
Take holy water, pray, fast	—	3 (3.7%)
No action	82 (67.8%)	40 (33.1%)

**Table 5:** Fisher's exact test result of association between student's health behaviour on use of prescribed medication and purpose of the medication intake.

	*N*	Completed medications	Did not complete	*p*
To prevent malaria	18	10 (55.6%)	8 (44.4%)	0.0186^*∗*^
To treat malaria	54	46 (85.2%)	8 (14.8%)	

Total	72	56 (77.8%)	16 (22.2%)	

^*∗*^Significant.

## Additional Points

*Recommendations*. (i) Health is fundamental right of all including pregnant women and their unborn babies; hence, there is a need for formal welfare programme that protect pregnant undergraduates and promote good pregnancy outcome. (ii) University health centres should be upgraded to provide basic antenatal care services and appropriate referral where necessary. (iii) The Tertiary Institution Social Health Insurance Programme should be reinforced and active Health Maintenance Organisations maintained at all university health centres to ensure the welfare of pregnant students and reduce their burden of out-of-pocket expenses. (iv) University policies should include regulations that allow departments and faculties to grant official permit to pregnant students to attend the antenatal clinic on their appointment days. (v) Health education on malaria infection causes, prevention and treatment, and dangers of drug abuse should, therefore, form part of orientation lectures for all freshmen. (vi) Billboards and posters with relevant information can be positioned at strategic places in the university as reminders on proper preventive health behaviours. Also, stakeholders in health can sponsor production of souvenirs with inscriptions about tips on malaria preventive behaviour written on them. Free ITN should be made regular component living-room material for all students. (vii) The study was only on resident students of universities. Further studies are needed to determine the degree of influence of accessibility of healthcare services on the student's overall health seeking behaviour for malaria prevention and treatment using pregnant students living outside the school environment.

## References

[1] World Health Organization (2013). *World Malaria Report 2013*.

[2] Federal Ministry of Health (2011). *National Policy on Malaria Diagnosis And Treatment*.

[3] Raimi O. G., Kanu C. P. (2010). The prevalence of malaria infection in pregnant women living in a suburb of Lagos. *African Journal of Biochemistry Research*.

[4] Centre for Disease Control and Prevention (2014). *Impact of malaria*.

[5] Schantz-Dunn J., Nour N. M. (2009). Malaria and pregnancy: a global health perspective. *Reviews in Obstetrics and Gynaecology*.

[6] Ezugbo-Nwobi I. K., Obiukwu M. O., Umeanato P. U., Egbuche C. M. (2011). Prevalence of malaria parasites among Nnamdi Azikwe University students and anti-malaria drug use. *African Research Review*.

[7] Adeyemo F. O., Makinde O. Y., Chukwuka L. O., Oyana E. N. (2013). Incidence of malaria infection among the undergraduates of university of Benin (Uniben), Benin City, Nigeria. *The Internet Journal of Tropical Medicine*.

[8] Faborode, M. The trouble with Nigerian universities. Punch Newspaper, 13/12/2012

[9] Aluko, B. Universities NEEDS Assessment Report Presentation to NEC November 2012. NigerianMuse, 2013

[10] Okojie, J. This is Your University! ThisDay Newspaper, 30/09/2013

[11] Ebehikhalu N. O., Dawam P. D. (2016). Inadequacy of Teaching and learning infrastructure: reason nigerian universities cannot drive innovations. *Journal of Educational Policy and Entrepreneurial Research*.

[12] Obiechina G. O., Ekenedo G. O. (2013). Factors affecting utilization of university health services in a tertiary institution in south-west Nigeria. *Nigerian Journal of Clinical Practice*.

[13] Afolabi M. O., Daropale V. O., Irinoye A. I., Adegoke A. A. (2013). Health-seeking behaviour and student perception of health care services in a university community in Nigeria. *Scientific Research*.

[14] Agomo C. O., Oyibo W. A., Anorlu R. I., Agomo P. U. (2009). Prevalence of malaria in pregnant women in Lagos, South-West Nigeria. *The Korean Journal of Parasitology*.

[15] World Health Organisation, WHO Malaria fact sheet. Available: http://www.ivcc.com/, (2011)

[16] World Health Organization, Malaria in pregnant women. Available: http://www.who.int/malaria/areas/high_risk_groups/pregnancy/en/, 2013

[17] Onyeneho N. G., Idemili-Aronu N., Igwe I., Iremeka F. U. (2015). Perception and attitudes towards preventives of malaria infection during pregnancy in Enugu State, Nigeria. *Journal of Health, Population and Nutrition*.

[18] Anumudu C. I., Adepoju A., Adediran M. (2006). Malaria prevalence and treatment seeking behaviour of young nigerian adults. *Annals of African Medicine*.

[19] Okwa O. O., Ibidapo A. C. (2010). IThe malaria situation, perception of cause and treatment in a nigerian university. *Journal of Medicine and Medical Sciences*.

